# Supplementary material for: Asynchronous magnetic resonance elastography: Shear wave speed reconstruction using noise correlation of incoherent waves
Source: Magn Reson Med. 2022 Oct 27;89(3):990–1001. doi: 10.1002/mrm.29502 (PMC9792433; doi:10.1002/mrm.29502)
Supplement: Supplementary file 1 — DATA S1 MATLAB code used to generate the numerical simulations of Figure 1 [file MRM-89-990-s002.zip › k-Wave/helpfiles/example_diff_focused_ultrasound_heating.html]

Heating By A Focused Ultrasound Transducer (k-Wave)


# Heating By A Focused Ultrasound Transducer

This example demonstrates how to combine acoustic and thermal simulations in k-Wave to calculate the heating by a focused ultrasound transducer. It builds on the Simulating Transducer Field Patterns and Using A Binary Sensor Mask examples.

- Open the file in the MATLAB Editor
- Run the file in MATLAB

## Contents

- Running the acoustic simulation
- Running the thermal simulation

## Running the acoustic simulation

In the previous examples, the temperature and heat source are defined using a Gaussian distribution. In this example, an acoustic simulation is first performed using `kspaceFirstOrder2D` to calculate the volume rate of heating from a focused ultrasound transducer in 2D. This is then used as an input to a thermal simulation to calculate the temperature map and ablated tissue volume. The volume rate of heat deposition can be approximated using the plane wave relationship `Q = alpha * p^2 / (c0 * rho0)`. For nonlinear simulations, `alpha` and `p` correspond to the absorption coefficient and pressure amplitude at each frequency harmonic.

For a linear simulation, the pressure amplitude `p` can be approximated by recording the maximum pressure once the simulation has reached steady state, i.e., by setting `sensor.record = {'p_max_all'}`, and `sensor.record_start_index` to a time step after the simulation has reached steady state. A more accurate estimate of the pressure amplitude can be obtained by setting the time step to give an integer number of time steps per period, and then recording the acoustic pressure over an integer number of periods in steady state. This allows the pressure amplitude at the driving frequency (and the harmonics if the simulation is nonlinear) to be obtained from the amplitude spectrum of the pressure time signals.

```
% calculate the time step using an integer number of points per period
ppw = medium.sound_speed / (freq * dx); % points per wavelength
cfl = 0.3;                              % cfl number
ppp = ceil(ppw / cfl);                  % points per period
T   = 1 / freq;                         % period [s]
dt  = T / ppp;                          % time step [s]
```

Here, the acoustic source is defined to be a focused transducer driven by a continuous wave sinusoid at 1 MHz with a surface pressure of 500 kPa. Note, the surface pressure is relatively high to achieve the required focal pressure to ablate the tissue. This is because the simulation is in 2D, and thus the focusing gain is much less than an equivalent simulation in 3D using a focused bowl transducer of the same radius and diameter.

## Running the thermal simulation

After the acoustic simulation, the acoustic pressure amplitude in steady state is extracted from the recorded pressure signals using `extractAmpPhase`. This extracts the amplitude from the frequency spectrum at the specified frequency. After calculating the acoustic pressure amplitude, the volume rate of heat deposition is calculated.

```
% convert the absorption coefficient to nepers/m
alpha_np = db2neper(medium.alpha_coeff, medium.alpha_power) * ...
    (2 * pi * freq).^medium.alpha_power;

% extract the pressure amplitude at each position
p = extractAmpPhase(sensor_data.p, 1/kgrid.dt, freq);

% reshape the data, and calculate the volume rate of heat deposition
p = reshape(p, Nx, Ny);
Q = alpha_np .* p.^2 ./ (medium.density .* medium.sound_speed);
```

The volume rate of heat deposition is assigned to `source.Q`, and `kWaveDiffusion` is used to simulate the temperature distribution and thermal dose with 10 seconds of heating, and 20 seconds of cooling. The resulting pressure, volume rate of heat deposition, temperature field, thermal dose, and ablation volume are shown below. Many extensions to this example are possible, for example, extending it to 3D, using heterogenous acoustic and thermal properties, and including perfusion (both background values and thermally significant individual vessels).
